# Cantilever Resin-Bonded Bridge as a Solution for the Replacement of a Single Missing Anterior Tooth: A Case-Based Review

**DOI:** 10.7759/cureus.93702

**Published:** 2025-10-02

**Authors:** Imane Hachami, Fatima Zahra Amessegher, Nezha Ben El Hammi, Houda Moussaoui

**Affiliations:** 1 Fixed Prosthodontics, Faculty of Dentistry of Casablanca, Hassan II University, Casablanca, MAR

**Keywords:** cantilver, minimally invasive, resin-bonded bridge, single edentulism, zirconia

## Abstract

Single-tooth edentulism in the anterior region presents a common clinical challenge. When implant placement is not feasible, the cantilever resin-bonded bridge (RBB), which relies on a single abutment tooth, emerges as a highly suitable alternative. This approach represents an evolution from the conventional metal-framed, double-wing RBB, offering a broader range of indications, from temporary prosthetic solutions to definitive anterior tooth replacement. With significant advantages, particularly in terms of tissue preservation, mastering and integrating this technique into contemporary clinical practice becomes essential.

This paper aims to address the management of single-tooth anterior edentulism with a cantilever resin-bonded bridge (RBB). For each clinical case, the diagnostic and therapeutic approach will be detailed. Two distinct workflows will be presented, one following a conventional approach and the other utilizing a digital workflow. Furthermore, special emphasis will be placed on gingival management, highlighting its crucial role in achieving optimal tissue integration and a harmonious esthetic outcome.

## Introduction

The situation of a single missing anterior tooth is frequently encountered in the daily practice of a dentist and represents a true challenge. It may concern the central incisor or the lateral incisor (particularly in cases of unilateral or bilateral agenesis) and requires immediate replacement for esthetic and functional reasons [1,2]. This challenge is further heightened by the significant psychological impact that anterior edentulism has on the patient [3].

Anterior tooth loss may result from dental caries, periodontal disease, dental trauma (most frequently affecting the central incisor), or iatrogenic complications such as external or internal root resorptions and perforations that ultimately require extraction.

The replacement of a single missing anterior tooth presents multiple therapeutic options, each with its own advantages and limitations.

While implants are widely considered the gold standard for single-tooth replacement, they may be contraindicated in cases of medical or anatomical limitations. Additionally, complications related to residual alveolar growth can occur, particularly in young patients, particularly patients with a hyperdivergent growth pattern [4]. This may result in an apical discrepancy between the implant-supported crown and adjacent natural teeth [5].

It is both reasonable and clinically significant to defer implant placement in the anterior region until adulthood (when bone growth is complete to ensure long-term stability and optimal esthetic outcomes [6].

In the interim, alternative treatment options such as auto-transplantation, orthodontic space closure, or resin-bonded fixed dental prostheses (RBFPDs) should be considered as long-term or provisional solutions until implant therapy becomes viable [7]. Conventional bridges are only justified when neighboring teeth already require extensive prosthetic or restorative treatment.

This article is structured as a case-based review. Two clinical cases are presented to demonstrate the management of single-tooth anterior edentulism with zirconia cantilever resin-bonded bridges (RBBs) in situations where implant therapy is not a viable option. These cases illustrate abutment selection criteria and soft tissue/gingival management, highlighting both the biological and mechanical considerations involved in this conservative treatment approach.

## Case presentation

Case 1 

A 22-year-old male patient presented requesting a fixed prosthetic replacement of his retained deciduous upper left lateral incisor. The tooth had previously undergone failed endodontic treatment due to extensive caries. The patient’s medical history was unremarkable. Extraoral examination revealed no abnormalities.

**Figure 1:**
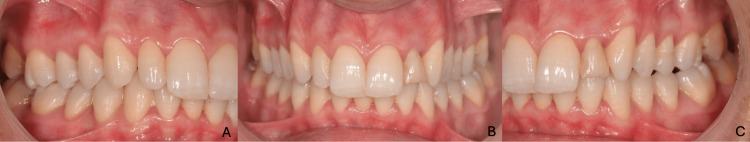
Intraoral initial views (A) Right lateral view; (B) frontal view; (C) left lateral view

**Figure 2:**
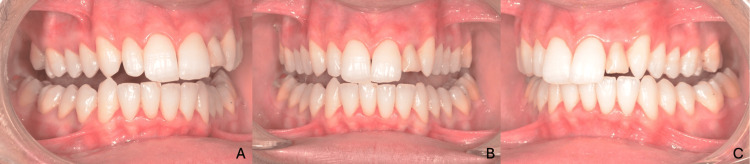
Dynamic occlusal analysis (A) Right lateral excursion showing canine guidance; (B) protrusive movement showing anterior guidance; (C) left lateral excursion showing the participation of tooth 21

Intraoral assessment showed good overall dental and periodontal health, with no signs of occlusal wear or parafunctional habits. However, the deciduous left lateral incisor appeared unesthetic and asymmetrical compared to its contralateral counterpart, exhibiting a vertical coronal fracture and multiple composite restorations. Occlusal analysis revealed a 4 mm overbite and a 3 mm overjet, with a right-sided Class I canine and molar relationship and a left-sided Class II canine and molar relationship (Figure 1). Dynamic occlusal assessment showed canine guidance on the right working side involving tooth 11, and disocclusion on the non-working side (Figure 2).

**Figure 3:**
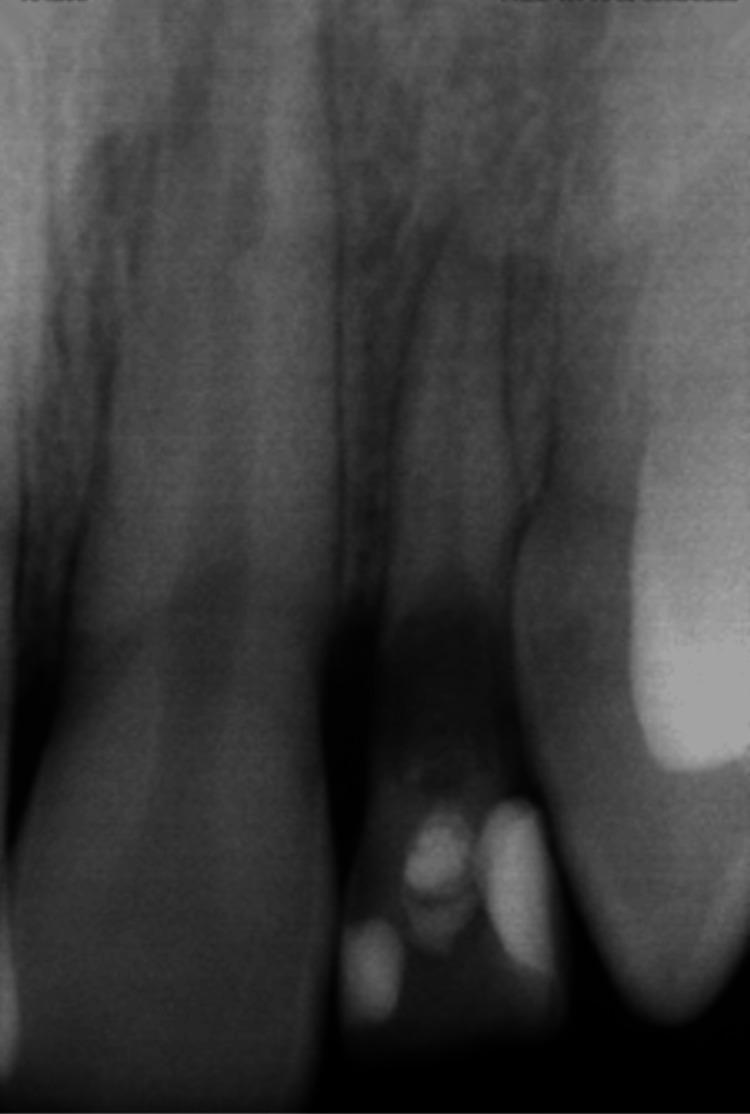
Initial radiograph of the retained deciduous upper left lateral incisor

**Figure 4:**
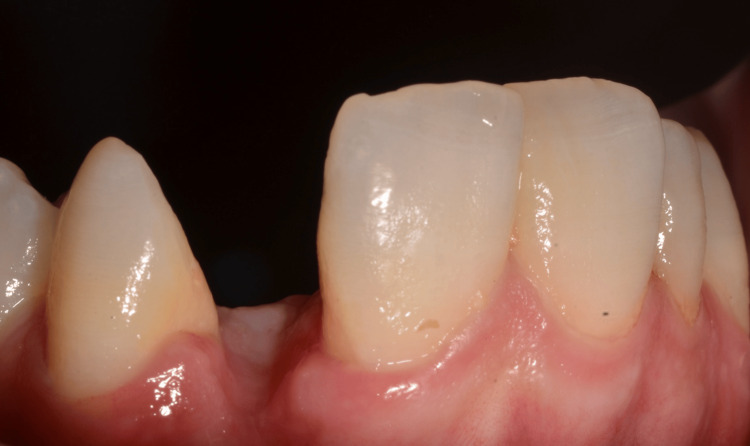
Post-extraction view of the edentulous site after removal of the retained deciduous upper left lateral incisor, showing the bone defect

Radiographic evaluation confirmed multiple coronal restorations along with advanced internal resorption, justifying the extraction of the deciduous tooth (Figures 3, 4).

The adjacent teeth (13 and 11) were structurally sound, with no signs of mobility and normal periodontal probing depths. Both abutment teeth provided sufficient sound enamel as the bonding substrate, with no existing restorations, ensuring favorable conditions for adhesive success. Tooth 21 showed slight labial inclination, and tooth 23 exhibited a tapered and asymmetrical morphology compared to its contralateral. Although orthodontic alignment could have improved the outcome, the patient declined any form of orthodontic treatment. Given his age and treatment preferences, implant placement was ruled out. Consequently, a zirconia cantilever RBB was selected as the most conservative and suitable treatment option.

Case 2 

A 23-year-old female patient was referred by her cardiologist for oral sanitation following a recent episode of infective endocarditis (IE). She had a prosthetic heart valve and was under anticoagulant therapy (Sintrom), placing her at high risk for IE.

**Figure 5:**
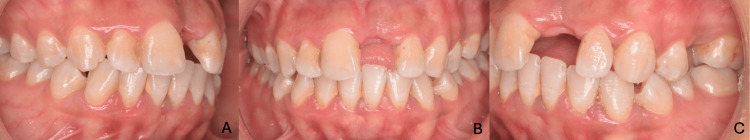
Initial intraoral views showing the anterior edentulous space (A) Right lateral view; (B) frontal view; (C) left lateral view

**Figure 6:**
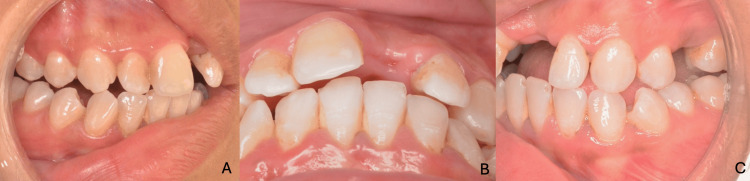
Initial functional analysis (A) Right lateral excursion; (B) protrusive movement; (C) left lateral excursion

The patient also reported a missing left central incisor due to trauma and previous direct restorations on both maxillary central incisors. Extraoral examination showed no abnormalities. Intraoral examination revealed multiple restorations on teeth 12, 11, and 21, as well as carious lesions on posterior teeth. Occlusal analysis showed a 3.5 mm overbite and a 5.5 mm overjet at tooth 11, with a right Class I canine and Class III molar relationship and a left Class I canine and molar relationship (Figure 5). Functional analysis revealed canine guidance on the working side and disocclusion on the non-working side (Figure 6).

**Figure 7:**
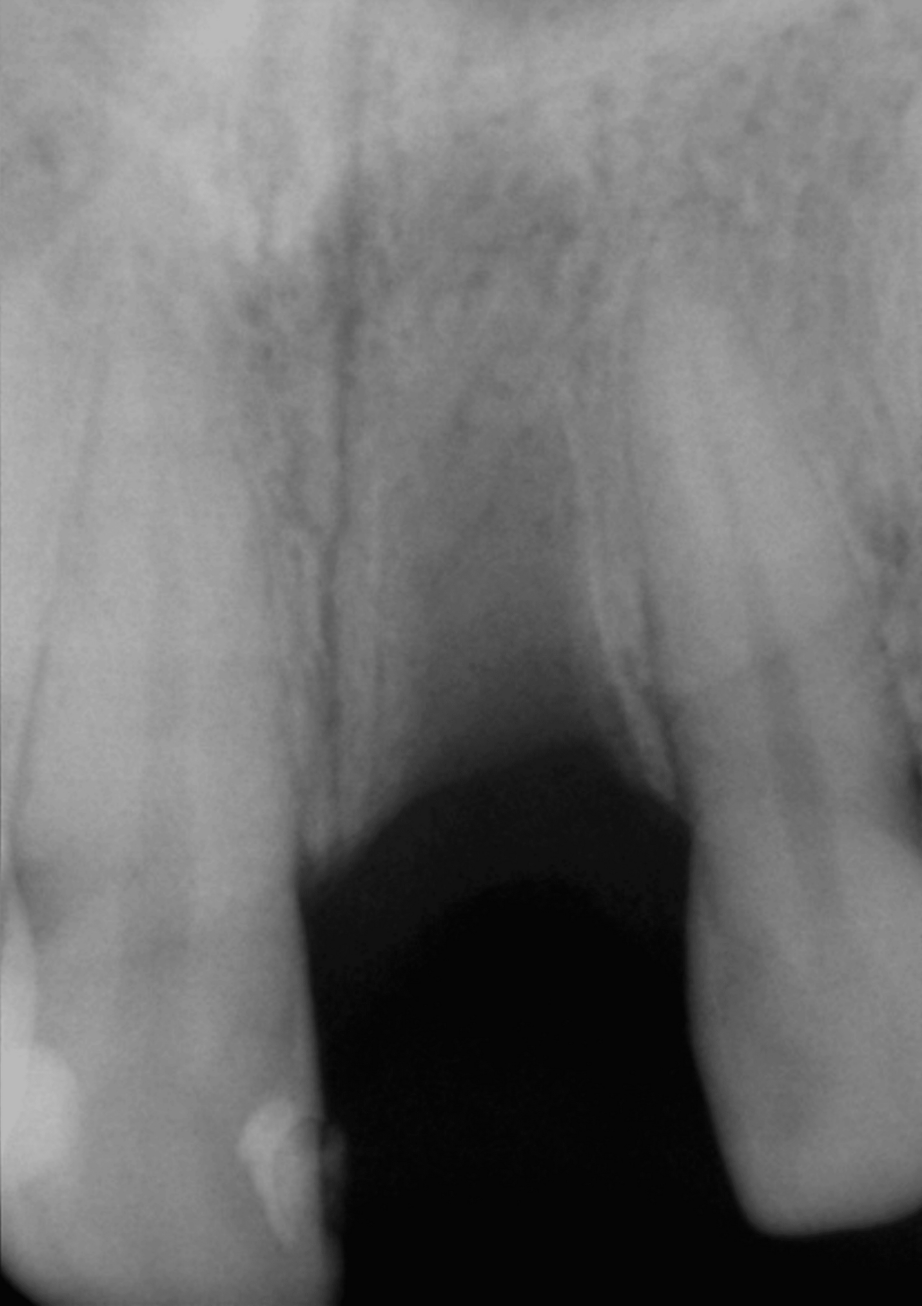
Initial periapical radiograph of the maxillary anterior region showing the edentulous space

Radiographic evaluation showed the restorations on the adjacent tooth (Figure 7).

While dental implant therapy has not been specifically documented in high-risk IE patients, it carries notable risks. Anticoagulant therapy may complicate surgical procedures, increasing the risk of hematoma and secondary infection. In the long term, peri-implant inflammation (mucositis or peri-implantitis) may also act as a potential infectious focus [8].

As a result, both the patient and her cardiologist ruled out implant placement. This left two viable treatment options: a conventional crown-retained fixed dental prosthesis or a cantilever RBB.

After removing the old restoration on the right central incisor and assessing the extent of the restoration as well as the remaining enamel, we were able to confirm the feasibility of a cantilever RBB as the treatment option.

After removing the old restoration on the right central incisor, the tooth was carefully evaluated. Periodontal probing depths were within normal limits, and the tooth exhibited no mobility. Assessment of the remaining structure confirmed the presence of sufficient sound enamel, providing an adequate bonding substrate despite the previous restoration. No secondary caries or cracks were detected. Based on these findings, the feasibility of a zirconia cantilever RBB was confirmed as the most appropriate treatment option.

Key pre-treatment considerations

Although a cantilever RBB is a relatively small prosthetic restoration, its success relies on thorough pre-treatment planning to ensure long-term stability and optimal function.

Before initiating the clinical steps, several critical factors must be carefully evaluated. Is there any contraindication to the use of an RBB? Which abutment tooth should be selected to support the retainer and the cantilevered pontic? What material should be chosen for the fabrication of the bridge?

Exclusion of Contraindications

The indication for a cantilever RBB requires a comprehensive assessment of several clinical factors that could either contraindicate its use or require prior orthodontic management. Malocclusion must be carefully considered since patients with deep bites, especially those with Class II Division 2 occlusion and steep anterior guidance, are generally poor candidates for RBB due to the excessive occlusal forces that can compromise restoration longevity. The structural integrity of the bonding surface is also critical; abutment teeth with large restorations or significant dentin exposure offer reduced bonding potential, thereby diminishing the predictability of long-term adhesive retention. Additionally, the edentulous space itself must be evaluated carefully, as cantilever RBBs are not appropriate when the gap is too wide or when a diastema must be preserved between the pontic and the abutment, given that this type of restoration does not allow for predictable or stable space maintenance under such conditions [9].

Selection of the Abutment Tooth

A crucial step in planning a cantilever RBB is the appropriate selection of the abutment tooth. This choice depends on the tooth being replaced and must be guided by both biomechanical and clinical criteria.

When replacing a central incisor, the contralateral central incisor is typically preferred as the abutment due to its symmetric positioning and similar biomechanical behavior, which reduces torque on the prosthesis.

When replacing a lateral incisor, either the adjacent central incisor or the canine can serve as the abutment. The decision requires careful evaluation of both intrinsic factors (tooth anatomy, bonding surface, periodontal support) and extrinsic factors (esthetics, occlusal dynamics, arch form) [10].

The central incisor is often preferred as the abutment tooth for supporting a lateral incisor pontic due to several advantages. It typically provides a greater connector height thanks to its tall proximal surface, allowing for a more robust and retentive design. Esthetically, the palatal wing is less visible on the central incisor than on the more curved and exposed palatal surface of the canine. Additionally, the central incisor offers a larger, flatter bonding surface that enhances adhesive retention and tends to present fewer occlusal interferences, as the palatal cingulum of the canine often occludes with the opposing canine and may require more reduction, compromising tooth structure. However, in specific cases, such as when the central incisor has compromised periodontal support or in patients with cleft lip and palate, the canine may serve as a better abutment choice. In these situations, the canine's wider proximal surface allows for a broader connector, and certain arch forms may offer improved esthetics through a naturally concealed embrasure. Furthermore, the curved palatal surface of the canine may resist dislodging forces more effectively, and its slightly thicker enamel can contribute to stronger adhesion [11].

The selection of the abutment should always be made on a case-by-case basis, considering periodontal status, quantity and quality of enamel available for bonding, alignment and position of the potential abutment, clinical crown height, and presence of existing restorations [12].

Consequently, in our clinical cases, the abutment selection was adapted to each scenario. The canine was chosen as the abutment tooth in the first case, while the right central incisor was selected in the second case.

Selection of Material

The choice of framework material for a cantilever RBB primarily concerns lithium disilicate and zirconia [13]. While both offer excellent esthetics and acceptable adhesive potential, a key distinction lies in their flexural strength. Lithium disilicate averages around 600 MPa, whereas 3Y-TZP zirconia can reach up to 1200 MPa.

From a bonding perspective, lithium disilicate offers a slight advantage, as it is an etchable ceramic and can be predictably bonded using hydrofluoric acid and silane. However, zirconia can also achieve durable adhesion, provided that a strict bonding protocol is followed. This will be detailed in a subsequent section.

This difference in mechanical behavior has crucial implications for cantilever restorations, where the connector often represents the weakest point. Its dimensions are critical to the long-term success of the prosthesis. Zirconia is able to maintain its mechanical integrity with relatively small connector dimensions of approximately 3 mm in height and 2 mm in width [14]. In contrast, lithium disilicate requires a larger connector, typically ranging between 12 and 16 mm², most often translated clinically into dimensions of about 3 mm by 4 mm. However, achieving such dimensions may not always be feasible in cases where the available space is limited [15,16].

Clinical approach

Although cantilever RBBs are minimally invasive, their success depends on rigorous execution of each clinical step.

Esthetic Analysis

The clinical workflow begins with a thorough esthetic assessment, both extraorally and intraorally. This initial step helps evaluate smile line, tooth proportions, midline harmony, and gingival architecture. An esthetic planning phase is then carried out, often involving a diagnostic wax-up on study casts or virtual models. This facilitates the anticipation and visualization of the outcome, guides clinical decision-making, and ensures alignment between patient expectations and prosthetic possibilities.

Ridge Preparation

One of the primary challenges in achieving the esthetic success of RBBs is ensuring a natural pontic emergence profile in harmony with the surrounding soft tissues. Therefore, a thorough muco-gingival analysis is essential to determine the periodontal biotype and assess the available height of keratinized gingiva. In many cases, prior modification of the edentulous ridge is required to create what is known as an oval pontic space.

The ovalization of the pontic site can be achieved through two main approaches. Additive surgery, which is recommended in cases with significant ridge deficiencies and involves connective tissue grafting to enhance soft tissue volume and contour, thereby improving the esthetic outcome. On the other hand, subtractive surgery is suitable when the ridge height and thickness are already sufficient. In such cases, the goal is to sculpt the most natural emergence profile for the pontic. This can be done using various instruments such as a round bur, laser, or electrosurgery to reshape the soft tissue, thus optimizing both the emergence profile and the presence of interdental papillae.

In addition to these surgical techniques, prosthetic conditioning plays a crucial role. This involves the use of an interim prosthesis, which may be a vacuum-formed stent incorporating a prosthetic or natural tooth, or a removable prosthesis. This device serves a dual purpose: it esthetically restores the missing tooth while simultaneously maintaining and shaping the gingival profile.

In both cases, the basal surface of the interim tooth is modified by applying a layer of flowable composite, giving it a convex shape in all dimensions. This facilitates soft tissue remodeling and optimizes the emergence profile. A healing period of approximately four weeks is recommended to ensure optimal tissue maturation before the final prosthetic rehabilitation [14].

**Figure 8:**
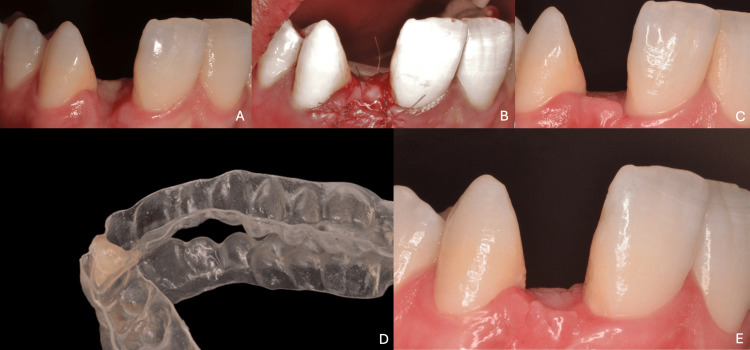
Gingival management of the ridge in the first case (A) Initial clinical situation showing ridge deficiency after extraction; (B) periodontal surgery with connective tissue grafting to augment ridge volume; (C) postoperative aspect after healing, showing improved ridge contour; (D) prosthetic stent with modified artificial tooth used for pontic site conditioning; (E) final aspect of the ridge with harmonious soft tissue architecture

For our clinical cases, following tooth extraction in the first case, a bone defect was observed that required periodontal reconstruction using connective tissue grafting. This procedure significantly improved the ridge’s width and height, and acceptable soft tissue conditioning, along with pontic site shaping, were achieved through the use of a prosthetic stent fitted with a modified prosthetic tooth. In the second case, medical contraindications prevented periodontal surgery, making it impossible to correct the ridge defect. This situation underscores the critical importance of gingival management in the successful fabrication of an RBB (Figure 8).

Abutment Tooth Preparation

**Figure 9:**
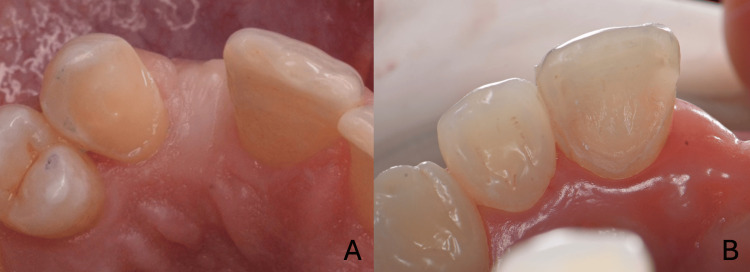
Preparation of the single abutment tooth for cantilever resin-bonded bridge (A) First clinical case; (B) second clinical case

Following pontic site management, attention is turned to the preparation of the abutment tooth, a critical phase for the success of an RBB. Although no universally standardized preparation design exists for zirconia-based RBBs, several fundamental principles must be respected to ensure optimal bonding and mechanical performance. The preparation should be minimally invasive and remain within the enamel whenever possible to maximize adhesion. A broad bonding surface should be provided, especially on the palatal face. Adequate space must be created to accommodate both the retainer wing and the connector. Material thickness must be respected to prevent fracture, and the design should facilitate the stable seating of the restoration during cementation. In both clinical cases, the palatal preparation of the abutment tooth followed a standardized and conservative approach, incorporating a supragingival chamfer of approximately 0.6 to 0.8 mm to preserve soft tissue health, a connector box on the proximal side facing the edentulous area to ensure sufficient material volume (3 mm height and 3 mm width) while remaining lingual to the contact point, palatal reduction of approximately 0.6 mm to provide the required thickness of zirconia and accommodate the retainer wing using a football-shaped diamond bur, and an occlusal ledge to extend the bonding surface without compromising incisal translucency or esthetics. In some cases, a small cementation pin is prepared to enhance positional stability during bonding; however, with the use of a silicone or printed repositioning key, this pin becomes optional (Figure 9) [13,16].

Impression Taking

**Figure 10:**
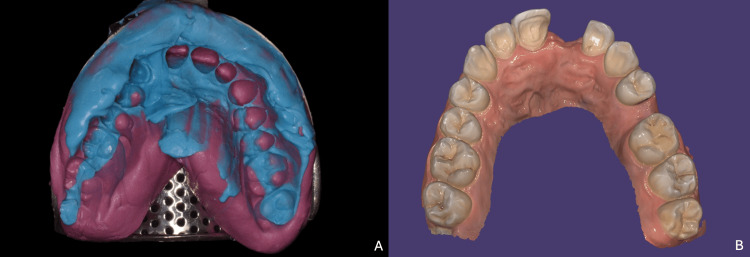
Impression procedures (A) Conventional dual-phase impression using putty and light-body silicone for the first clinical case; (B) digital impression obtained with an intraoral scanner for the second clinical case

In the first case, a conventional impression was taken using a dual-phase (putty and a light-body) polyvinyl siloxane single-stage technique, while in the second case, a digital impression was performed using a 3D intraoral scanner, Aoralscan 2 (SHINING 3D Tech. Co., Ltd., Hangzhou, China). Regardless of the impression technique used, certain preliminary requirements must be met. It is essential to ensure that the abutment and adjacent teeth are clean and dry, and if gingival inflammation is present, it should be managed through oral hygiene instruction and professional cleaning prior to the impression appointment. Additionally, a gingival retraction cord should be used if necessary to improve the visibility of the preparation margins. Finally, a full-arch impression should be performed rather than a sectorial one to ensure accurate occlusal adjustment in both static and dynamic function (Figure 10).

Laboratory Communication 

**Figure 11:**
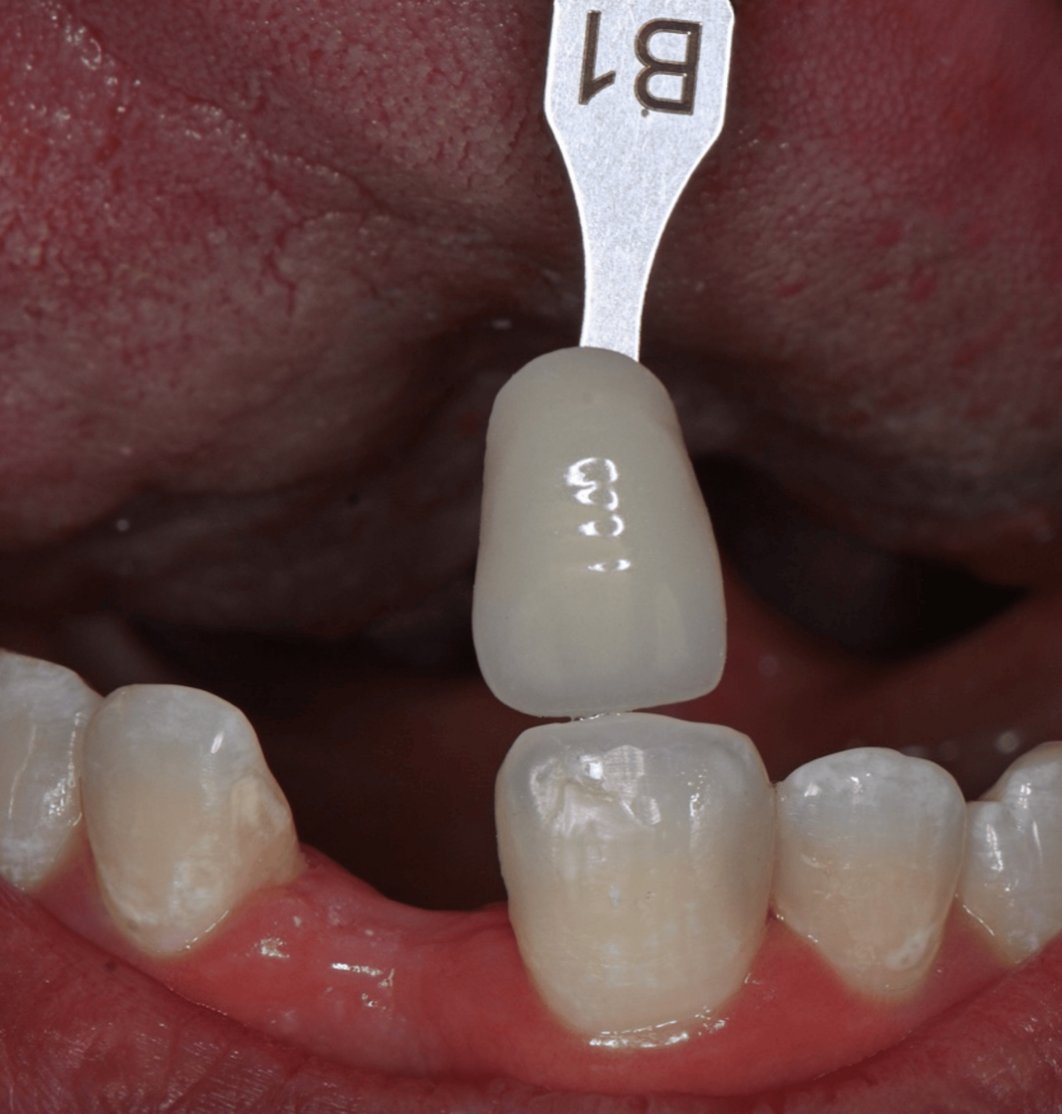
Shade selection using the VITA Classical Shade Guide

As with any esthetic restoration, effective communication with the dental laboratory is crucial for the success of an RBB. Detailed and accurate information allows the dental technician to meet both functional and esthetic requirements. In both clinical cases, high-resolution clinical photographs were provided, including images with the selected shade tab using the VITA Classical Shade Guide [17]. These visuals were accompanied by a clear description of the restorative material, connector design, and desired emergence profile. This collaborative approach with the dental ceramist ensured precise shade matching, improved contour design, and contributed to achieving an optimal esthetic outcome tailored to each patient's individual morphology and expectations (Figure 11).

Try-In 

**Figure 12:**
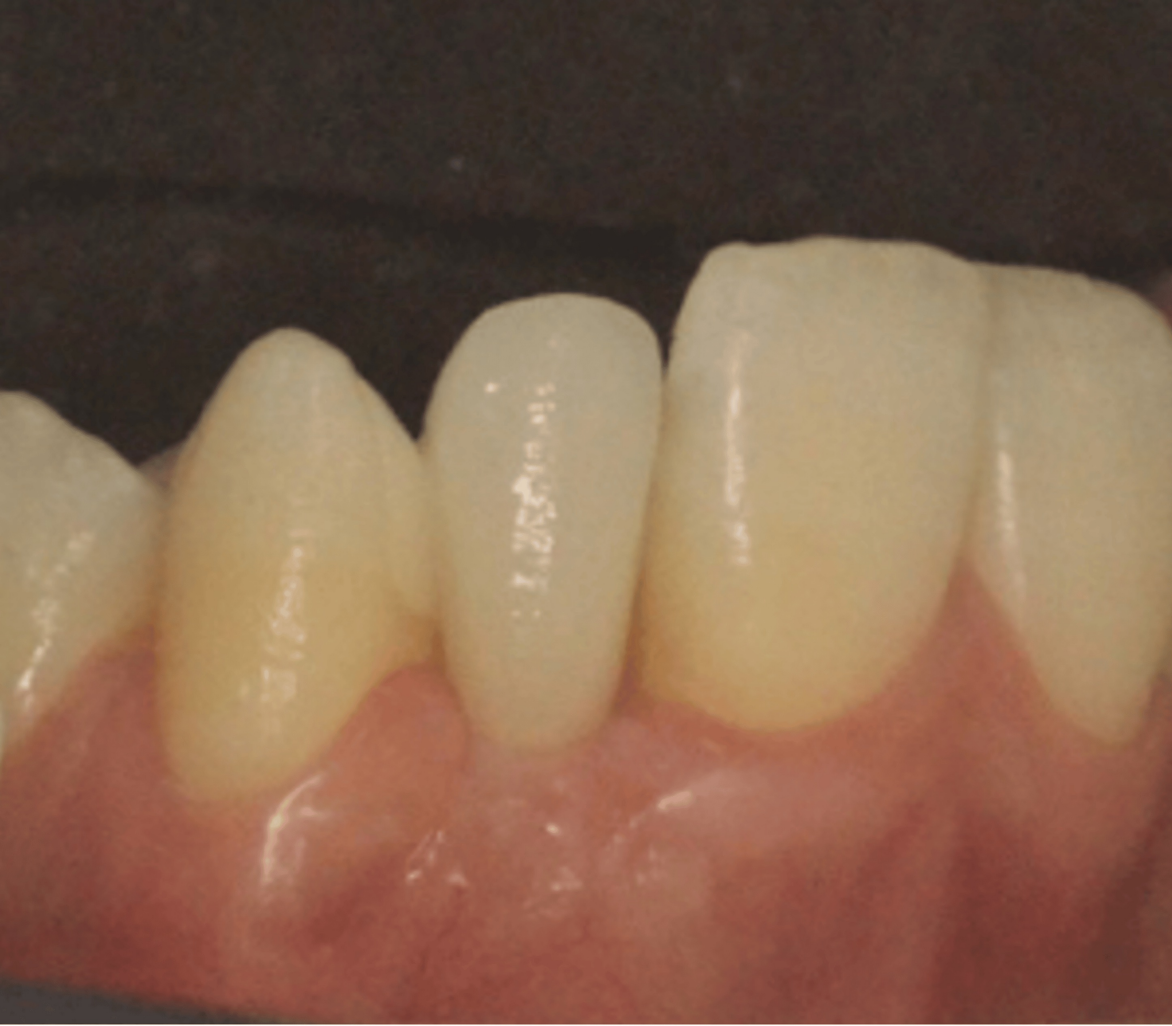
Picture with a polarizing filter for shade correction of the first case

The try-in appointment is a critical stage in the clinical workflow, allowing for comprehensive evaluation of the restoration before final bonding. At this stage, several elements are carefully assessed, including the fit of the retainer wing on the abutment tooth, the shade integration with adjacent teeth, the pontic morphology and its emergence profile relative to the gingiva, the accuracy of proximal contact points, and the occlusion in both static and dynamic movements. To facilitate temporary placement during this phase, we used TempBond™ Clear (Kerr Corporation, Brea, CA, USA) as recommended by Matthias Kern [14]. This transparent provisional cement maintains a gel-like consistency after light-curing, providing adequate retention while remaining easily removable with a dental probe. In the first case, the initial shade of the restoration was not entirely satisfactory, and to improve the esthetic outcome, additional photographs were taken using a polarizing filter to eliminate light reflections and accurately capture tooth hue and chroma. These images were then forwarded to the dental laboratory for shade correction via surface staining, which resulted in improved esthetic integration (Figure 12).

*Bonding* 

The long-term success of zirconia-based RBBs relies heavily on a meticulously executed bonding protocol, particularly due to zirconia’s inherent resistance to conventional etching techniques. For both cases, we followed the APC protocol, developed by Markus Blatz, which is widely recognized as the gold standard for adhesive cementation of zirconia restorations [18]. The APC protocol consists of three steps: air abrasion, primer application, and composite resin cement application.

**Figure 13:**
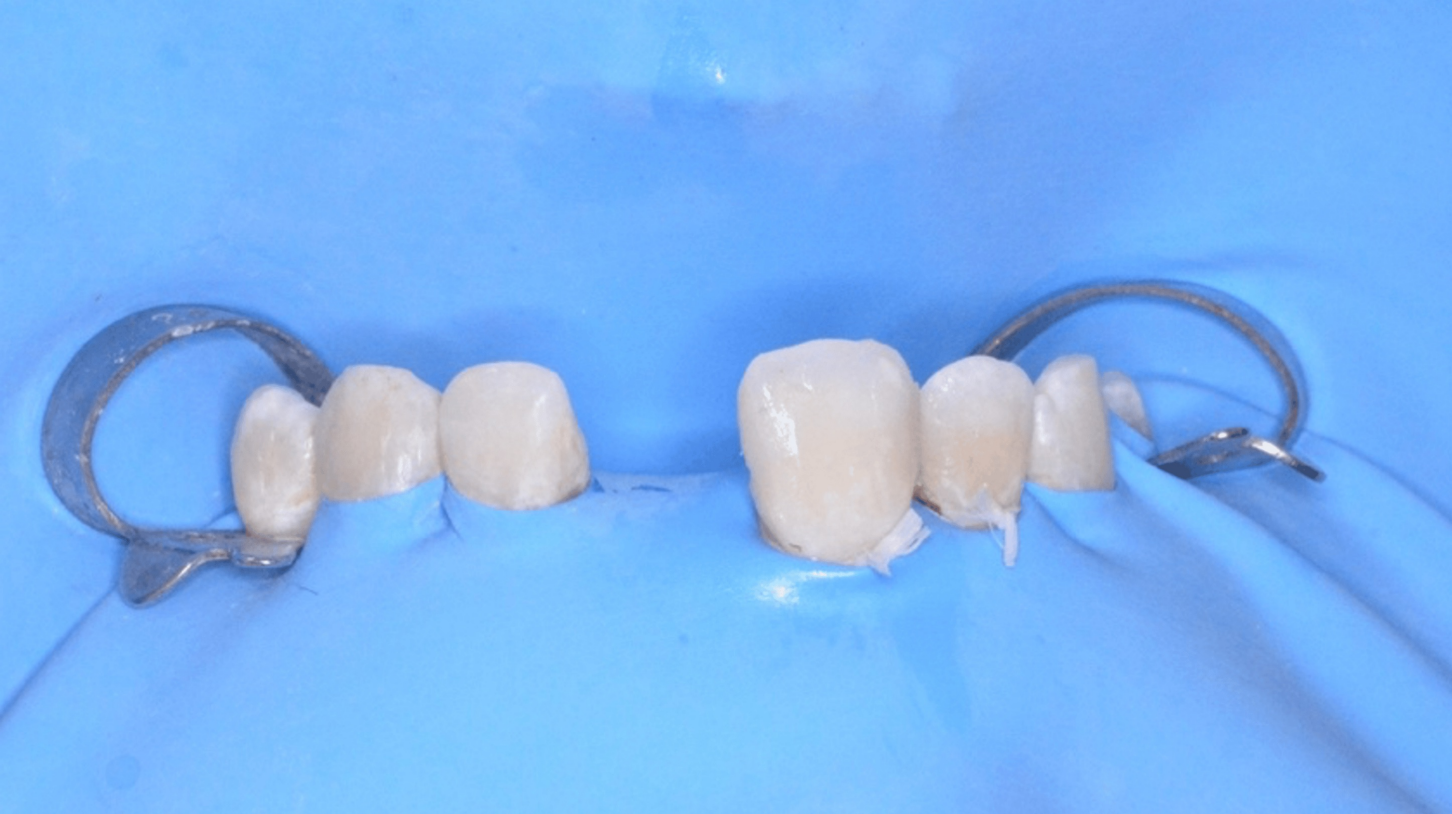
Rubber dam isolation of the abutment tooth in the second clinical case

A rubber dam was placed to isolate the working field, ideally from tooth 14 to 24, to ensure a clear and dry operating area (Figure 13). However, in RBBs, the so-called “trampoline effect” caused by the dam beneath the pontic may hinder proper seating of the bridge. To counter this, perforations should be sufficiently spaced to allow stress-free insertion of the prosthesis, and a bonding key (also called positioning key), previously fabricated in the laboratory, was used to stabilize the bridge during cementation and ensure precise seating of the retainer onto the abutment tooth.

Air-Particle Abrasion

**Figure 14:**
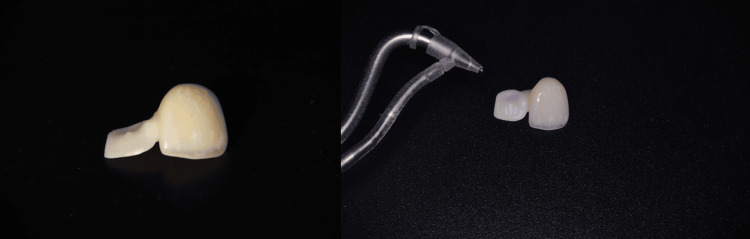
Air-abrasion of the second cantilever resin-bonded bridge

Immediately after try-in, the intaglio surface of the zirconia restoration was cleaned using micro air-abrasion: 52 µm aluminum oxide, at 2-3 bars of pressure, from a 10 mm distance, for 10 seconds. No rinsing was performed afterward, only a gentle air spray. This was carried out using the Aquacare system (Velopex International, Saint Cloud, FL, USA). This step enhances micromechanical retention and prepares the zirconia surface for chemical bonding (Figure 14).

Primer Application

**Figure 15:**
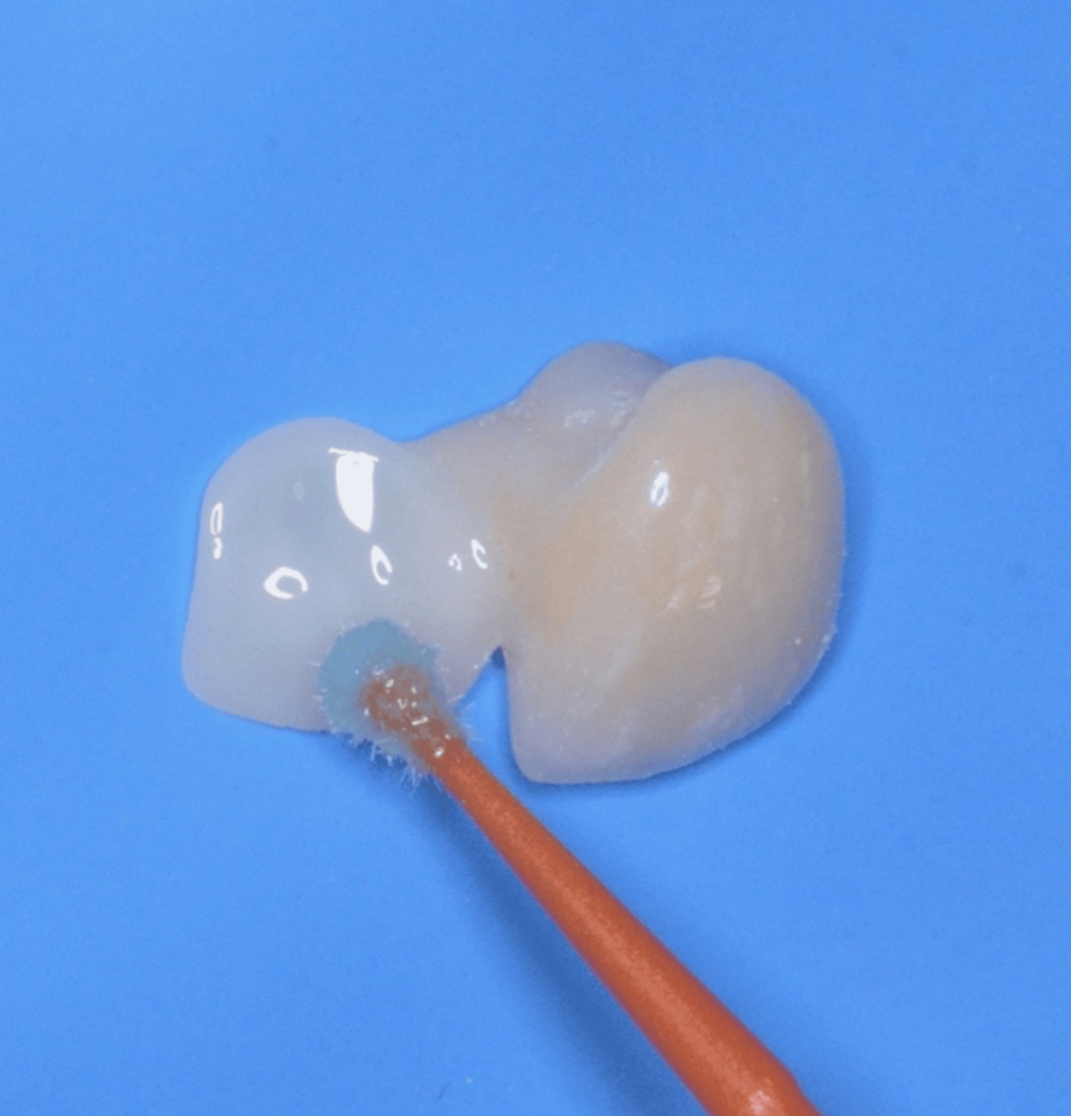
Application of the 10-MDP primer on the second cantilever resin-bonded bridge 10-MDP: 10-methacryloyloxydecyl dihydrogen phosphate

Chemical adhesion to zirconia is made possible by using a primer containing the 10-MDP monomer (methacryloyloxydecyl dihydrogen phosphate). In our cases, Monobond N (Ivoclar Vivadent AG, Schaan, Liechtenstein) was applied directly after air-abrasion (Figure 15). It combines both 10-MDP and silane coupling agent for improved bond strength. The primer was left to air-dry while tooth surface conditioning was initiated.

Composite Resin Cement Application

**Figure 16:**
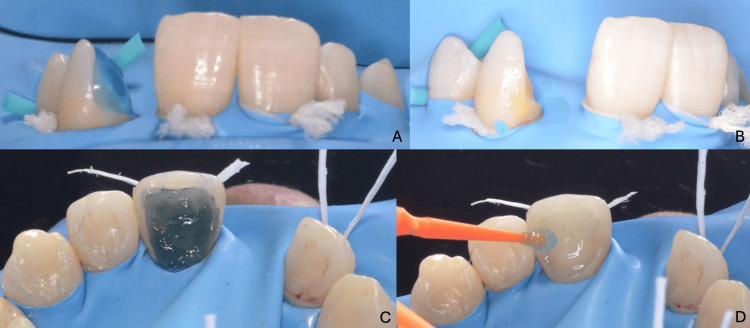
Conditioning of the abutment enamel surface prior to bonding (A, B) First clinical case: enamel etching under rubber dam isolation and application of the adhesive; (C, D) second clinical case: enamel etching under rubber dam isolation and application of the adhesive

For the final cementation, we used Estecem Plus (Tokuyama Dental Corporation, Tokyo, Japan), a dual-cure, radiopaque adhesive resin cement with excellent esthetic and handling characteristics. To prepare the abutment enamel surface, the surface was brushed with fluoride-free pumice to eliminate plaque or debris, then rinsed and dried. Enamel margins were selectively etched with 35-37% phosphoric acid for 10-15 seconds, then rinsed for 15 seconds and lightly air-dried. Palfique Universal Bond A and B (Tokuyama Dental Corporation, Tokyo, Japan) were mixed and applied to both the etched enamel and the sandblasted internal surface of the zirconia restoration. Weak air was applied until the adhesive stopped moving, followed by mild drying (Figure 16). 

**Figure 17:**
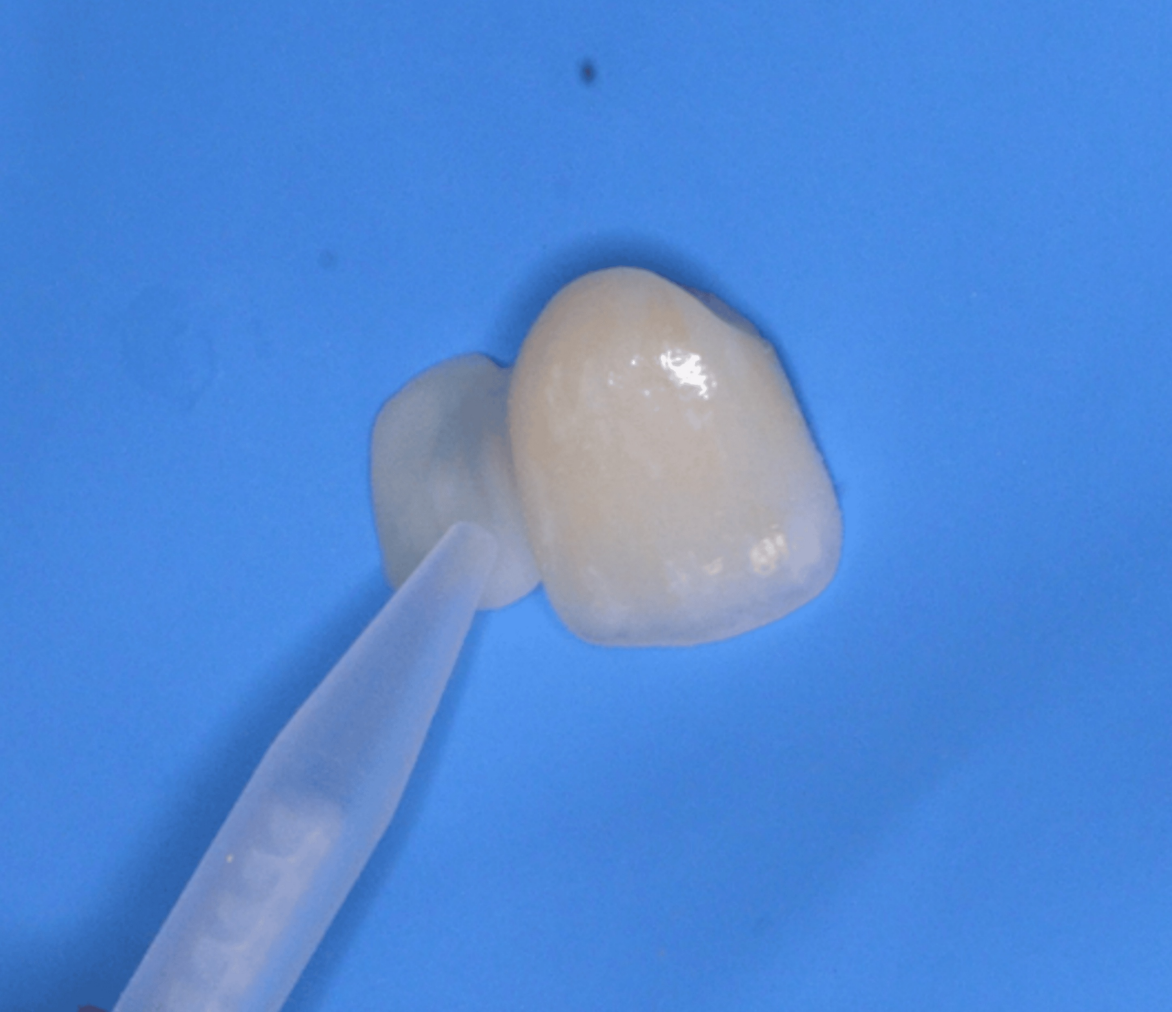
Application of the resin cement to the sandblasted second resin-bonded bridge

**Figure 18:**
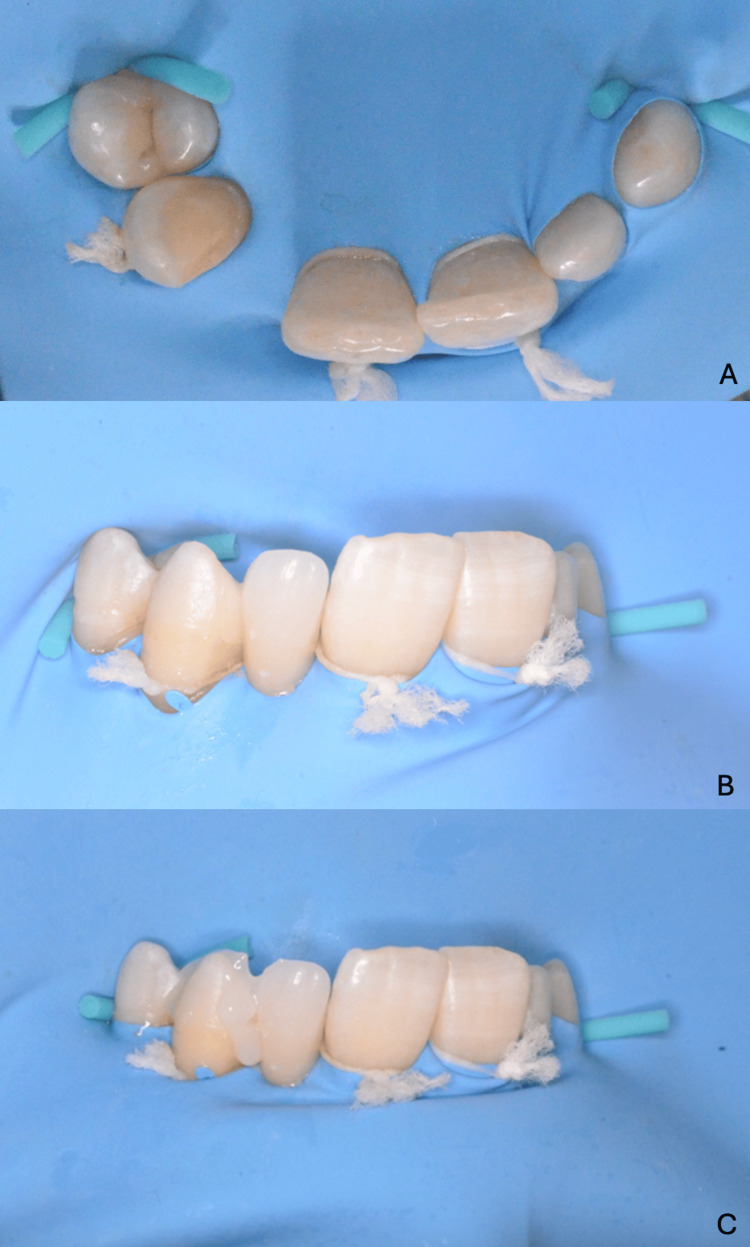
Seating and bonding procedure of the first resin-bonded bridge (A) Rubber dam isolation and placement before bonding; (B) try-in and positioning of the restoration under the rubber dam; (C) application of glycerin gel prior to final polymerization

The bonding procedure began with the application of the mixed resin cement to the bonding surface of the zirconia retainer. The bridge was then carefully seated using the bonding key to ensure precise positioning. Light-curing was performed for two to four seconds to reach the gel phase, during which the excess cement was gently removed. Each margin was then light-cured for 20 seconds, followed by an eight-minute self-curing period. Finally, glycerin gel was applied to prevent the formation of an oxygen-inhibited layer before the final cure (Figures 17, 18).

Occlusal Check

**Figure 19:**
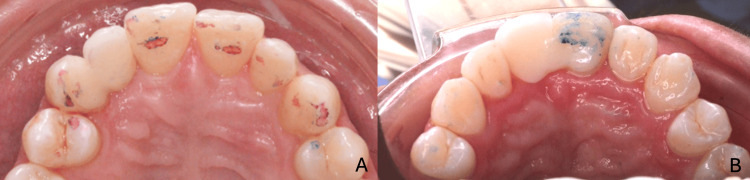
Dynamic occlusal check after bonding (A) First clinical case with the first resin-bonded bridge (RBB); (B) second clinical case with the second RBB

The final clinical step, often underestimated, is the verification of occlusion, which plays a critical role in the long-term stability of a cantilever RBB. In static occlusion, contact points may be shared between the retainer wing and the pontic, ensuring balanced load distribution. However, in dynamic occlusion, particularly during lateral or protrusive movements, the pontic must remain out of contact to prevent excessive functional stress that could lead to debonding or fracture (Figure 19).

**Figure 20:**
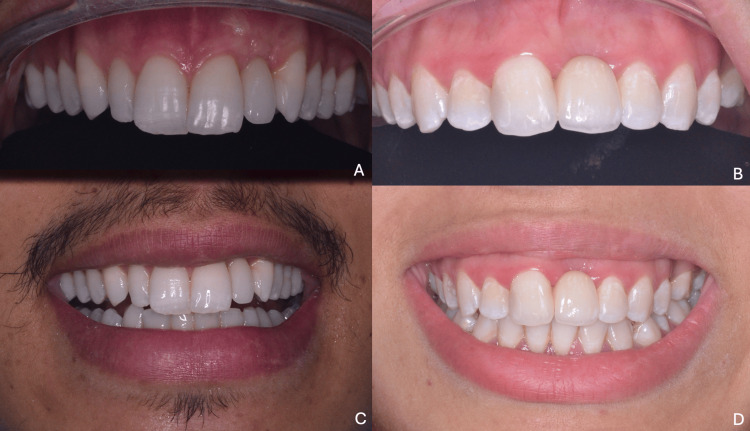
Final clinical outcomes (A, C) First clinical case: intraoral and extraoral smile views; (B, D) second clinical case: intraoral and extraoral smile views

Occlusal adjustments should remain minimal, especially at the connector and wing levels, as over-reduction could compromise the mechanical integrity of the zirconia. However, if occlusal modifications are required, the adjusted zirconia surfaces must be meticulously polished. This prevents microcrack propagation and minimizes abrasion of opposing dentition, thereby preserving the restoration and the natural teeth (Figure 20).

## Discussion

Since their introduction in the 1960s, RBBs were initially developed as minimally invasive prosthetic options aimed at replacing single missing teeth while preserving tooth structure and offering reversibility. The first generations, such as Rochette bridges and later Maryland bridges, were based on metal frameworks bonded to enamel surfaces. However, despite their conservative approach, these early designs showed disappointing long-term survival rates [14].

Limitations of Dual-Wing Designs

One of the main complications observed with dual-wing RBBs was partial debonding, typically affecting only one of the two retainers. Often unnoticed by patients, this unilateral loss of adhesion allowed micro-movements at the interface, which over time led to secondary caries beneath the detached wing. This complication was especially common when one abutment tooth, usually the one with less physiological mobility, remained bonded, causing stress concentration and failure on the other. The difference in the physiological axis of mobility between incisors and canines could explain such failures. Interestingly, clinical experience demonstrated that removing the detached wing from a failing two-wing RBB could significantly extend the longevity of the restoration, sometimes by several years [16].

Shift Toward Cantilever Design 

The development of cantilever single-wing RBBs represented a paradigm shift. These restorations solved many of the limitations associated with dual-retainer bridges, particularly in young patients or implant-contraindicated cases. Cantilever RBBs proved to be both esthetic and biologically conservative, with survival rates comparable to implant-supported restorations. Matthias Kern of the University of Kiel pioneered this concept in the 1990s by validating the use of ceramic cantilever RBBs and systematically demonstrating that single-wing RBBs yielded significantly better clinical outcomes than dual-wing designs [14]. In his landmark 2005 study, Kern reported a five-year survival rate of 92.3% for cantilever RBBs, compared to 73.9% for two-wing designs over the same period [19]. This reinforced the reliability of the monolithic design, especially when combined with strict bonding protocols and appropriate case selection [19].

Material Evolution and Clinical Implications

Initially made from metal frameworks, RBBs have since evolved to incorporate high-performance ceramics, notably zirconia and lithium disilicate, both offering superior esthetic and mechanical properties [16].

For zirconia frameworks, Klink and Hüttig (2016) reported a success rate of 82.4% over three years, with a 100% survival rate [20]. A longer-term study by Kern et al. showed a 92% success rate over 10 years, and a 98.2% survival rate, with debondings primarily following traumatic events and all successfully rebonded [21].

While zirconia offers excellent mechanical strength and predictable rebonding in case of failure, it remains more susceptible to debonding compared to disilicate. On the other hand, lithium disilicate, though more bondable, may, in rare cases, suffer connector fractures, which may require complete replacement.

MDP-based primers have become a cornerstone in establishing durable adhesion to zirconia. Because zirconia lacks a glassy phase, conventional etching and silanization are ineffective. The phosphate group of 10-MDP forms a stable chemical bond with the zirconium oxide surface, while the methacrylate group polymerizes with resin-based cements. This dual interaction significantly improves shear bond strength and resists hydrolytic degradation over time. When used in combination with airborne-particle abrasion, MDP primers create a synergistic effect that ensures both high initial bond strength and long-term stability, which is crucial for the clinical success of zirconia RBBs [22].

Synthesis and Clinical Relevance

The clinical performance of cantilever RBBs depends on three pillars: careful case selection and occlusal analysis, material choice based on prosthetic space and bonding needs, and strict adherence to modern adhesive protocols. In this context, zirconia cantilever RBBs, as used in our two cases, provide an excellent balance between esthetics, longevity, and repairability, especially when coupled with rigorous protocols like the APC system. These restorations, once considered temporary solutions, are now recognized as long-term therapeutic options, particularly suitable for young patients, medically compromised individuals, or implant-restricted zones.

## Conclusions

Cantilever resin-bonded bridges (RBBs), particularly when fabricated in zirconia and bonded with rigorous adhesive protocols, offer a conservative, reliable, and esthetically satisfying solution for the replacement of a single anterior tooth. The two clinical cases present and highlight the importance of thorough case selection, occlusal and esthetic analysis, and pre-prosthetic soft tissue management to ensure long-term success. Advances in adhesive dentistry and ceramic materials have significantly improved the predictability and durability of this minimally invasive option.

While not universally applicable, zirconia cantilever RBBs represent a valuable alternative to implant therapy, especially in medically compromised or young patients, when handled with clinical rigor and in close collaboration with the dental technician. Nevertheless, it should be noted that long-term survival data for anterior zirconia cantilever RBBs remain less abundant compared to metal frameworks, and clinical outcomes are highly technique-sensitive, particularly regarding occlusion and enamel-only bonding.
